# Antioxidative Defense, Suppressed Nitric Oxide Accumulation, and Synthesis of Protective Proteins in Roots and Leaves Contribute to the Desiccation Tolerance of the Resurrection Plant *Haberlea rhodopensis*

**DOI:** 10.3390/plants12152834

**Published:** 2023-07-31

**Authors:** Katya Georgieva, Gergana Mihailova, Liliana Gigova, Antoaneta V. Popova, Maya Velitchkova, Lyudmila Simova-Stoilova, Máté Sági-Kazár, Helga Zelenyánszki, Katalin Solymosi, Ádám Solti

**Affiliations:** 1 Institute of Plant Physiology and Genetics, Bulgarian Academy of Sciences, Academic Georgi Bonchev Str., Building 21, 1113 Sofia, Bulgaria; gmihailova@bio21.bas.bg (G.M.); gigova01@gmail.com (L.G.); lsimova@mail.bg (L.S.-S.); 2 Institute of Biophysics and Biomedical Engineering, Bulgarian Academy of Sciences, Academic Georgi Bonchev Str., Building 21, 1113 Sofia, Bulgaria; popova@bio21.bas.bg (A.V.P.); mayav@bio21.bas.bg (M.V.); 3 Department of Plant Physiology and Molecular Plant Biology, Institute of Biology, ELTE Eötvös Loránd University, Pázmány Péter Sétány 1/C, H-1117 Budapest, Hungary; sagi.kazar.mate@ttk.elte.hu (M.S.-K.); helga.zelenyanszki@ttk.elte.hu (H.Z.); adam.solti@ttk.elte.hu (Á.S.); 4 Doctoral School of Biology, Institute of Biology, ELTE Eötvös Loránd University, Pázmány Péter Sétány 1/C, H-1117 Budapest, Hungary; 5 Department of Plant Anatomy, Institute of Biology, ELTE Eötvös Loránd University, Pázmány Péter Sétány 1/C, H-1117 Budapest, Hungary; katalin.solymosi@ttk.elte.hu

**Keywords:** antioxidant enzymes, drought stress, non-enzymatic antioxidants, nitric oxide, photosynthesis, protective proteins, root anatomy

## Abstract

The desiccation tolerance of plants relies on defense mechanisms that enable the protection of macromolecules, biological structures, and metabolism. Although the defense of leaf tissues exposed to solar irradiation is challenging, mechanisms that protect the viability of the roots, yet largely unexplored, are equally important for survival. Although the photosynthetic apparatus in leaves contributes to the generation of oxidative stress under drought stress, we hypothesized that oxidative stress and thus antioxidative defense is also predominant in the roots. Thus, we aimed for a comparative analysis of the protective mechanisms in leaves and roots during the desiccation of *Haberlea rhodopensis*. Consequently, a high content of non-enzymatic antioxidants and high activity of antioxidant enzymes together with the activation of specific isoenzymes were found in both leaves and roots during the final stages of desiccation of *H. rhodopensis*. Among others, catalase and glutathione reductase activity showed a similar tendency of changes in roots and leaves, whereas, unlike that in the leaves, superoxide dismutase activity was enhanced under severe but not under medium desiccation in roots. Nitric oxide accumulation in the root tips was found to be sensitive to water restriction but suppressed under severe desiccation. In addition to the antioxidative defense, desiccation induced an enhanced abundance of dehydrins, ELIPs, and sHSP 17.7 in leaves, but this was significantly better in roots. In contrast to leaf cells, starch remained in the cells of the central cylinder of desiccated roots. Taken together, protective compounds and antioxidative defense mechanisms are equally important in protecting the roots to survive desiccation. Since drought-induced damage to the root system fundamentally affects the survival of plants, a better understanding of root desiccation tolerance mechanisms is essential to compensate for the challenges of prolonged dry periods.

## 1. Introduction

In the 21st century, evidence becomes clear on climate change, the primary challenge for humankind presently and in the future. Since extremities in the weather such as prolonged drought are among the greatest threats to agriculture, the predicted increasing periods of intense and extended drought as a consequence of global warming will have a deep impact on food production [1]. The vegetative tissues of higher plants, including crops, are sensitive to water deficiency: depending on the species, loss of 40–70% of total water content leads to permanent damage or death of the tissues [2]. Thus, prolonged drought stress and critical dehydration ultimately reduce the crop yield. However, resurrection plants are able to survive up to 95% loss of tissue water content and thus survive prolonged drought periods, whereas upon rehydration they recover to full metabolic activity [3]. Therefore, they are an optimal model to study and understand vegetative desiccation tolerance. Such understanding may contribute to the breeding of crops with improved tolerance [4]. A considerable number of studies pointed out that foliar tissues of resurrection plants are able to tolerate desiccation through a special set of mechanisms, including the alteration of the metabolism and the biosynthesis of novel antioxidants to minimize free radical-induced damages, subcellular reorganization in order to minimize mechanical stress associated with turgor loss, and the accumulation of specific proteins, disaccharides, and other hydrophilic metabolites to maintain the structure and the operation of cell constituents [5,6,7,8,9]. Since, in photosynthetically active tissues, suppression of the photosynthetic functions and avoiding light-induced damages are primarily challenges to be resolved in the desiccating foliar tissues for effective survival, the majority of studies on resurrection plants focused on the protective mechanisms that protect mesophyll cells. Nevertheless, in the survival of individual plants, responses of root tissues that are equally or even better exposed to desiccation have remained an unexplored field.

In foliar desiccation tolerance studies, homoiochlorophyllous resurrection plants that maintain the structure of the photosynthetic apparatus during dehydration are generally applied. Since, in these plant taxa, chlorophyll molecules retained in the desiccated stage could be sources for harmful singlet oxygen production in mesophyll cells, protection mechanisms, including rapid repair of the photosynthetic apparatus upon rehydration, are required. It has been proposed that switching off photosynthesis in homoiochlorophyllous plants is likely to be a programmed process that involves specific protective mechanisms [10]. While the production of reactive oxygen species associated with photosynthesis is reduced during desiccation in photosynthetically active tissues, the oxidative stress associated with metabolic processes of mitochondria and particularly peroxisomes is exacerbated with acute water loss [11]. Thus, oxidative stress is not exclusively associated with the photosynthetic apparatus even in the foliage. Moreover, the upregulation of antioxidant activity is generally acknowledged as one of the key protective mechanisms induced during desiccation. It has been shown that resurrection plant individuals can survive desiccation without damage to the leaves as long as their foliar antioxidant system is functional [12]. In contrast to desiccation-sensitive plants, desiccation-tolerant plants can maintain foliar antioxidant activity in the desiccated state [5]. Accumulation of non-enzymatic antioxidants and enhanced activity of antioxidant enzymes have been observed during desiccation [6,7]. Although a considerable variation was detected in the antioxidant responses of different resurrection taxa under desiccation and rehydration [13], their longevity in the desiccated state was generally dependent on antioxidative protection [14]. Nevertheless, these statements were not taken into a more general context, i.e., how the protective mechanisms of the root system contribute to the individual’s survival [15,16]. *Haberlea rhodopensis* Friv. is a poikilohydric, perennial, herbaceous plant that, as a preglacial relict taxon, primarily inhabits shady, northern limestone slopes of relatively high humidity in mountainous regions of the Balkan Peninsula, South-East Europe. It is a generally applied homoiochlorophyllous resurrection plant model, in which antioxidative protection was previously found to be crucial to the survival of foliar desiccation [7]. Although foliar responses of *H. rhodopensis* to desiccation are well documented, the contribution of the root system to desiccation tolerance is much less studied.

Regarding the desiccation tolerance mechanisms in roots, studies on the amount of carbohydrates reported an increased sucrose content in the root system upon the desiccation of *Craterostigma plantagineum*, *Pitcairnia burchellii*, and *Tripogon loliiformis* [17,18,19]. Dehydrated roots of *Tripogon loliiformis* were found to contain more sucrose and trehalose-6-phosphate compared to shoots, and the plant continued to use roots as a carbohydrate sink during drought stress, activating the bidirectional sugar transporter *SWEET* genes to translocate sucrose from shoots to roots [19]. Investigation on glycerolipids present in root and leaf tissues during the desiccation of *Xerophyta humilis* revealed an increase in species containing polyunsaturated fatty acids and a decrease in saturated species [20]. Our previous studies showed that the plasticity of adaptation in *H. rhodopensis* leaves and roots during extreme water stress conditions is different [21,22]. In general, roots are more sensitive and respond faster to water stress than leaves. The specific leaf area is reduced by 60% as a result of severe desiccation, whereas the root area decreased by 40%. A linear relationship between root respiration and water content is established. In addition, analysis of *H. rhodopensis* leaves and roots revealed that jasmonic acid, along with and even earlier than abscisic acid, serves as a signal that triggers the response of this resurrection plant to desiccation [23]. In the biosynthesis of abscisic acid, nitric oxide (NO) generation in root tips was found to be an early response in *Triticum aestivum* [24]. Thus, NO production in plants is among the first reactions to drought [25]. NO contributes to the regulation of multiple cell processes, including antioxidative defense and the formation of reactive nitrogen species, but also the synthesis of defense proteins [26]. Antioxidative protection is an important process in roots. Investigating the activity of the antioxidant enzymes superoxide dismutase (SOD), catalase (CAT), ascorbate peroxidase (APX), and glutathione reductase (GR), as well as ascorbate and glutathione content in the roots of *Xerophyta viscosa* during desiccation, root tissues appear to retain their antioxidant potential during drying for use in recovery upon rehydration, similar to leaf tissues but also to leaves of other resurrection taxa [27]. To understand the overall strategy of plant desiccation tolerance, holistic studies focusing simultaneously both on root and leaf systems are required.

Although the photosynthetic apparatus in leaves contributes to the generation of oxidative stress under drought stress, we hypothesized that oxidative stress and thus antioxidative defense is also predominant in the roots. In order to get a holistic view, we performed a comparative analysis of the desiccation tolerance mechanisms in leaves to those operating in the root system in the homoiochlorophyllous model plant *H. rhodopensis*. The anatomy and ultrastructure of the roots and NO production of the root tips were studied. Antioxidant capacity in leaves and roots from the same plant was assessed and compared by measuring the total antioxidant activity, free-radical scavenging activity, content of non-enzymatic antioxidants, and activity of antioxidant enzymes. The alterations in stress-induced proteins (dehydrins, ELIPs, sHSPs) in leaves and roots during desiccation and after recovery on rehydration were compared. We have characterized, for the first time, the protective mechanisms of the root system in the Gesneriaceae resurrection plant model *H. rhodopensis*.

## 2. Results

### 2.1. Changes in the Water Content of Leaves and Roots during Desiccation 

Control leaves (LC) of *H. rhodopensis* were characterized by higher water contents compared to control roots (RC) (Table 1). The first sampling point during dehydration was at a moderately dehydrated state, which is very characteristic of *H. rhodopensis* with its wilting drooping leaves becoming elastic. In moderately dehydrated plants, the WC of leaves (LD1) and roots (RD1) was reduced by 56% and 70%, respectively. In the D2 stage of desiccation, the WC of leaves (LD2) and roots (RD2) was equal and further water loss led to a faster drop of WC in leaves than in roots (LD3 and RD3, respectively). The leaves and roots were completely desiccated in the D4 stage (Figure S1B). *H. rhodopensis* leaves and roots in well-hydrated and air-dry states were compared in Figure S1. During the rehydration of plants, leaves (LR) and roots (RR) recovered their WC. 

### 2.2. Structural Changes in Root Tissues during Desiccation

**Figure 1:**
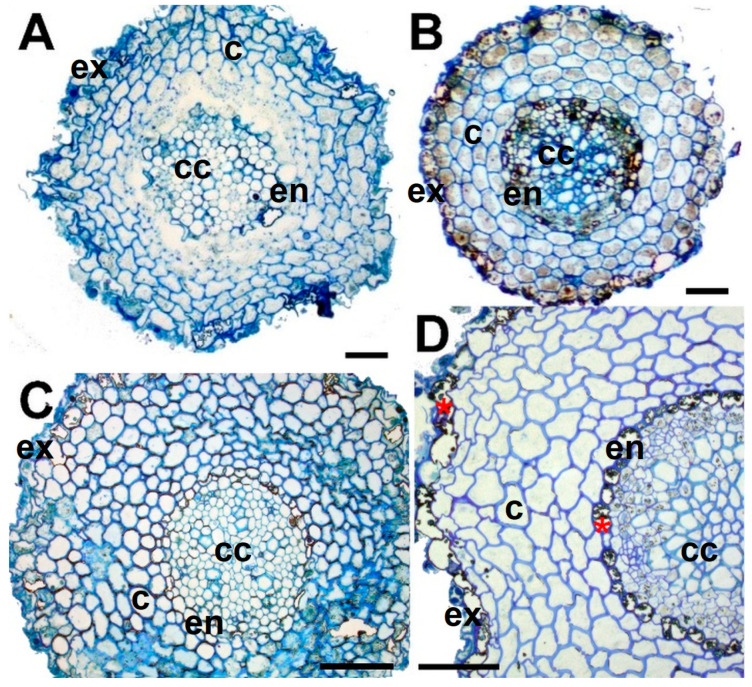
Root anatomy in the well-hydrated stage (RC) and after different extents of desiccation according to Table 1. Trans sections of roots at (**A**): RC, (**B**): RD2, (**C**): RD3, (**D**): RD4. Scale bar: 50 µm. Cell layers are labelled: ex–exodermis; c–cortex; en–endodermis; cc–central cylinder. Asterisks (in red) indicate dense accretions in exodermis and endodermis layers.

**Figure 2:**
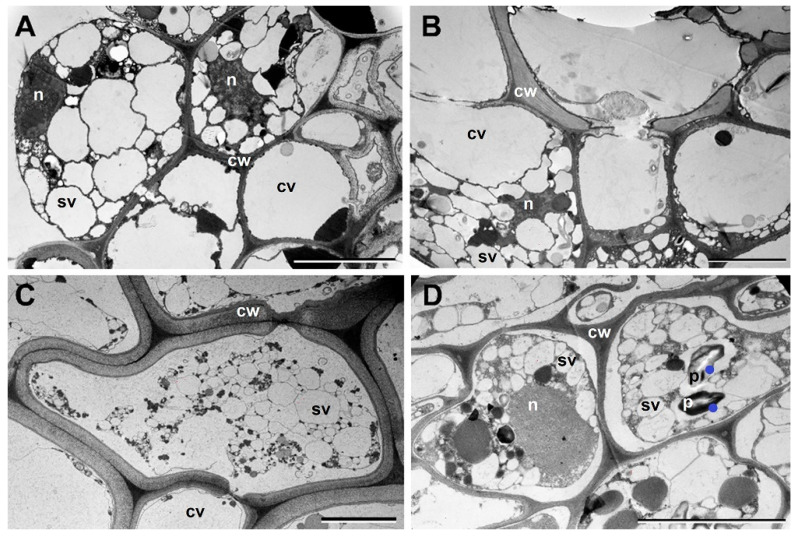
Ultrastructure of root cells (R) in the well-hydrated stage (RC) and after complete desiccation (RD4) (**A**,**B**): RC, (**C**,**D**): RD4. (**A**,**C**): cortex cells, (**B**,**D**): central cylinder cells. Scale bar: 5 µm. Cell compartments are indicated: cw–cell wall; n–nucleus; p–plastid; cv–central vacuole; sv–small vacuole. Dots (in blue) indicate starch accumulation.

We observed no striking structural alterations in the roots upon dehydration; however, a massive accumulation of dense accretions occurred in the cytoplasm of the exodermis and endodermis layers of the cortex of the desiccated roots (Figure 1). Transmission electron microscopy showed that these cells contained electron-dense dark deposits (not shown). Careful comparison of the root ultrastructure showed that in addition to heterochromatinous nuclei, several small vacuoles were already present in both cortex and central cylinder cells in the RC stage (Figure 2A,B). However, the number of vacuoles seemed to increase upon desiccation in both regions, but especially in the cortex (Figure 2C,D). Interestingly, we have also clearly observed the accumulation of starch (in amyloplasts) in the cells of the central cylinder of desiccated roots.

### 2.3. Nitric Oxide Production in the Root Tips

**Figure 3:**
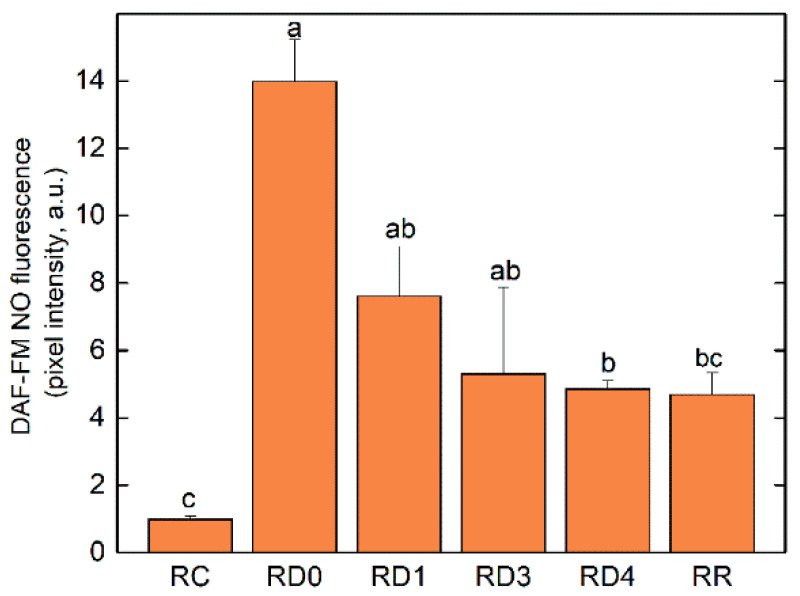
Average intensity of the NO signal in the apical zone of roots, based on the DAF-FM NO fluorescence intensity corrected on the basis of the non-stained roots. Root samples (R) were taken from the well-hydrated (RC), dehydrated to different extents (RD0–RD4), and fully rehydrated (RR) stages, according to Table 1. RD0 is a supplementary sample that was taken at a WC of 1.92 ± 0.05 g H_2_O g^−1^ DW as for an intermediate sample at the RC–RD1 transition.

The presence of nitric oxide (NO) in *H. rhodopensis* roots in the course of desiccation and after rehydration was detected based on the autofluorescence signal of 4-amino-5-methylamino-2′,7′-difluorofluorescein (DAF-FM) NO conjugates after trapping NO. The intensity of the autofluorescence signal (Figure S2) showed a remarkable increase upon the initiation of desiccation (Figure S2B). However, the elevated NO signal decreased during the more intensive loss of water content (Figure S2C–E) and remained relatively stable upon rehydration (Figure S2F). The intensity of the NO signal in the D0 stage corresponded to a WC of 1.92 ± 0.05 (g H_2_O g^−1^ DW) of the roots and showed a 10-times higher signal intensity compared to the well-hydrated control (Figure 3), whereas the decline in the NO signal in the later stages of desiccation showed a rather gradual profile. Indeed, it is important to note that the NO signal remained significantly higher in the air-dry stage compared to the well-hydrated control. Upon rehydration, a further decrease was measured in NO signal intensity, but during the applied rehydration period, the NO level did not fully restore the signal intensity measured in the well-hydrated control.

### 2.4. Antioxidant Activity in Leaves and Roots during Desiccation

#### 2.4.1. Antioxidant Capacity

**Figure 4:**
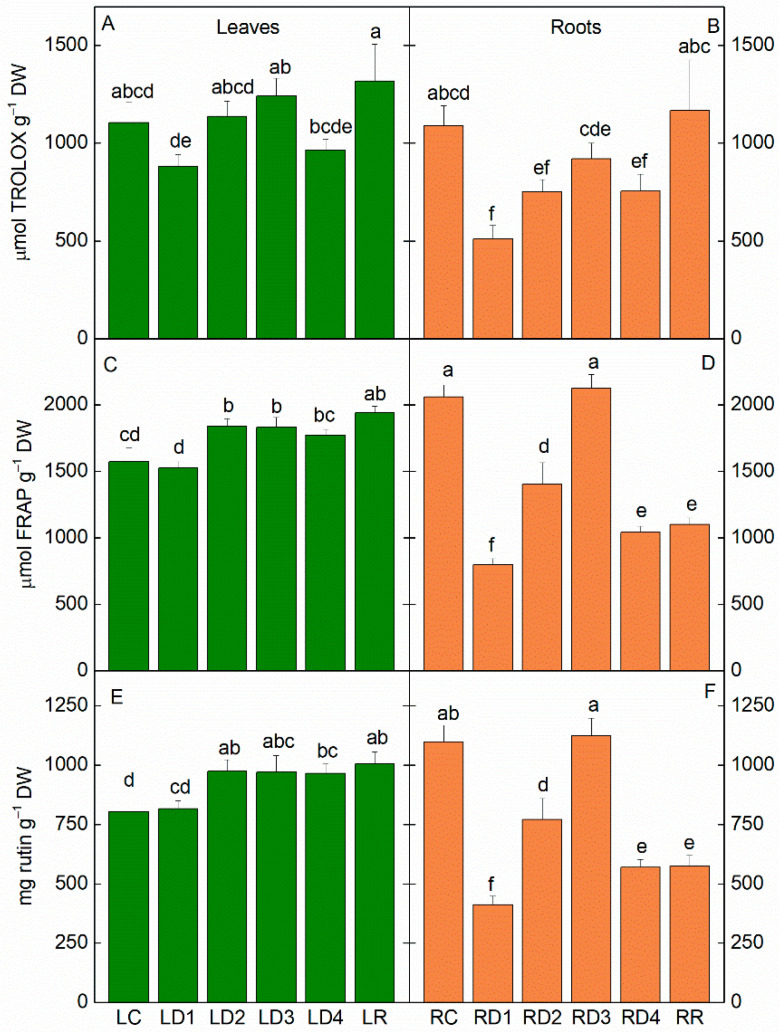
Free radical scavenging activity (**A**,**B**), total antioxidant activity (**C**,**D**), and total flavonoid content (**E**,**F**) in leaves (L; **A**,**C**,**E**) and roots (R; **B**,**D**,**F**) of *H. rhodopensis* in well-hydrated (LC, RC), dehydrated to different extents (LD1–LD4; RD1–RD4), and fully rehydrated (LR, RR) *H. rhodopensis* samples, according to Table 1.

The antioxidant capacity in *H. rhodopensis* leaves and roots in the course of desiccation was assessed based on the free-radical scavenging activity, total antioxidant activity, and flavonoid content. Despite a slight decline in the antiradical scavenging activity in the D1 and D4 states in the leaves, the values remained high upon desiccation. Similar changes were observed in the roots, but the observed decrease in antiradical activity was statistically significant and lower compared to the leaves (Figure 4B). The antioxidant activity and flavonoid content were close to those of the controls in moderately dehydrated leaves, but significantly increased with further water loss (*p* ≤ 0.05, Figure 4C,E) and remained high after rehydration. Similar to antiradical activity, antioxidant activity and flavonoid content in roots declined in RD1, then increased, reaching a maximum in RD3. Antioxidant activity was 50% lower in desiccated roots (RD4) and after rehydration (Figure 4D,F) than in the controls.

#### 2.4.2. Changes in Non-Enzymatic Antioxidants during Desiccation

**Figure 5:**
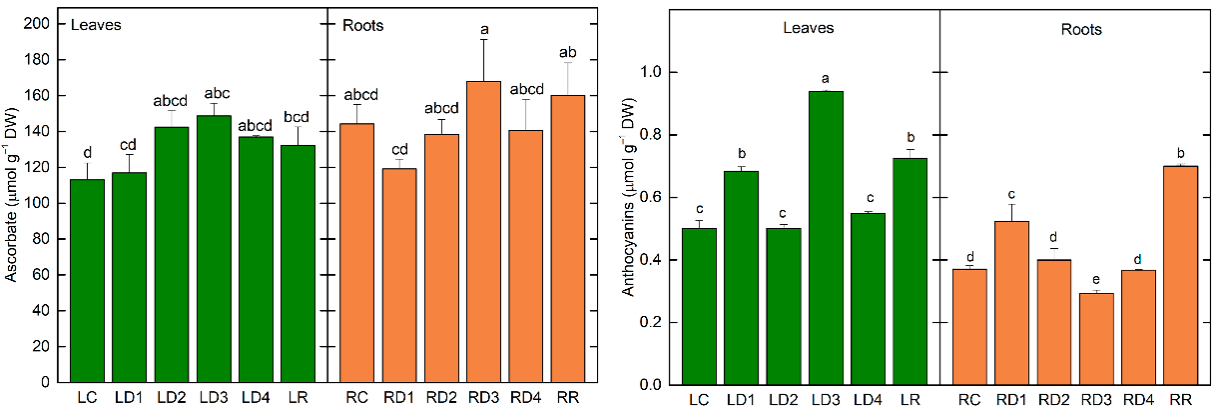
Changes in ascorbate (Asc, (**left**)) and anthocyanins content (**right**) in leaves and roots of *H. rhodopensis* in well-hydrated (LC, RC), dehydrated to different extents (LD1–LD4; RD1–RD4), and fully rehydrated (LR, RR) *H. rhodopensis* samples, according to Table 1.

The ascorbate (Asc) content in leaves increased in LD2 and remained high during further desiccation and after 6 days of rehydration, indicating its protective role under these conditions. Asc content in well-hydrated control roots was higher compared to leaves (Figure 5; 144 and 113 µmol g^−1^ DW, respectively), reaching the highest value in RD3 as determined for flavonoids (Figure 4F). Similar to leaves, the Asc content remained high in dry roots and following rehydration (Figure 5).

In fully hydrated plants of *H. rhodopensis*, the anthocyanins content of leaves was around 26% higher than in roots (Figure 5; 0.500 µmol g^−1^ DW and 0.370 µmol g^−1^ DW, respectively). During the initial period of desiccation, leaves (LD1) and roots (RD1) responded to decreased water availability in a similar manner. The content of anthocyanins was elevated by about 40% in leaves and roots in comparison with controls (LC and RC). Further dehydration resulted in values comparable with the respective control, valid for leaves (LD2) and roots (RD2). The most strongly expressed difference in anthocyanins content between leaves and roots was detected in D3, when the anthocyanin amount in the leaves was elevated by 88%, while a 20% decline was detected in the roots. At the highest degree of water loss, both leaves (LD4) and roots (RD4) showed values similar to the fully hydrated plants. After the recovery of plants from desiccation, leaves and roots responded with a significant increase in the anthocyanins content (by 45% for leaves and a much stronger 90% in roots). 

**Figure 6:**
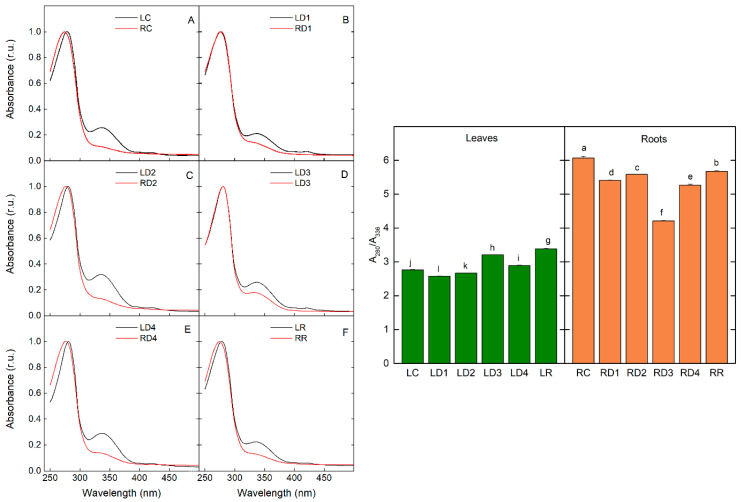
Comparison of absorption spectra of acidified methanolic extracts (**left**) and dependence of the ratio of absorbance at 280 nm to absorbance at 336 nm (A_280_/A_336_) (**right**) of leaves and roots of *H. rhodopensis* in well-hydrated ((**A**); LC, RC), dehydrated to different extents ((**B**–**E**); LD1–LD4; RD1–RD4), and fully rehydrated ((**F**); LR, RR) *H. rhodopensis* samples, according to Table 1. Absorption spectra were measured as indicated in Materials and Methods and normalized at the low-wavelength peak (Band II, 280 nm).

The absorption spectra of leaf and root extracts from *H. rhodopensis* plants desiccated to different WC are presented in Figure 6. While in leaf extracts the absorbance band at 336 nm was a well-expressed maximum, for root extracts this maximum was presented as a shoulder with lower amplitude. The amplitude of both bands changed with the progress of desiccation, for leaves as well for roots. For the control, D1 and D2, the ratio of amplitudes of Band II to Band I (A_280_/A_336_) almost did not change and followed a similar tendency for leaves and roots. The values of the A_280_/A_336_ ratio for roots were considerably higher as the intensity of Band I in root extracts was very low. The only difference was observed for LD3 and RD3, where the ratio increased for leaves and decreased for roots. After rehydration, the ratio increased for both leaf and root extracts, and was more pronounced in the latter.

#### 2.4.3. Isoenzyme Patterns and Relative Total Activity of Antioxidant Enzymes

**Figure 7:**
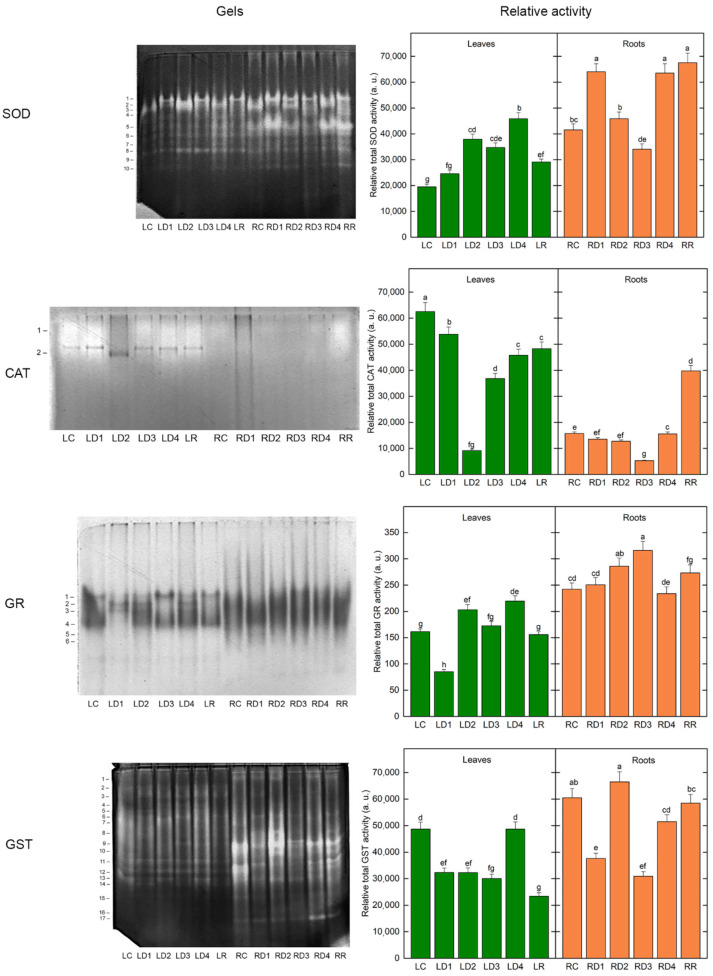
Isoenzyme pattern (**left**) and relative total activity (**right**) of superoxide dismutase (SOD), catalase (CAT), glutathione reductase (GR), and glutathione S-transferase (GST) in leaves and roots of well-hydrated (LC, LR), dehydrated to different extents (LD1–LD4; RD1–RD4), and fully rehydrated (LR, RR) *H. rhodopensis* samples, according to Table 1. The isoenzymes are numbered from cathode to anode. The total activity is expressed as a sum of the values (in arbitrary units) for integrated optical density of the respective isoenzymes.

In all *H. rhodopensis* protein extracts, ten SOD isoenzymes (numbered in the order of increasing electrophoretic mobility) were resolved in the gels after performing native PAGE (Figure 7 left). During desiccation, as well as under subsequent rehydration, a significant increase (ranging from 26 to 153%, *p* ≤ 0.05) in the relative total activity of SOD in leaves was observed (Figure 7 right) due to the activation of almost all enzyme isoforms, albeit to various degrees (Figure 7 left). In roots, SOD was significantly activated at RD1 and RD4 (both by 54%), mainly due to the upregulation of isoform 5 (by 130 and 123%, respectively) and isoform 1 (by 149 and 43%, respectively) (Figure 7). The roots’ enzyme activity was higher than the control in the rehydrated plants as well, with all isoenzymes (except for isoenzyme 2) participating in the increase. Although the total SOD activity in RD3 was significantly lower and in RD2 was similar to the control level, some specific isoenzymes were upregulated. In the case of the RD3 sample, the activities of isoenzymes 3 and 4 were increased by about 150% each, while the activity of isoenzymes 1 and 10 was elevated by about 84 and 36%, respectively, in the RD2 sample compared to the control plants. Overall, SOD in the roots showed significantly higher activity than in the leaves (by 113% for the control and by 160, 21, 0, 39, and 132% for each of the subsequent treatments). Only in the D3 state SOD was equally active in both plant organs (Figure 7, LD3 and RD3).

Staining for CAT revealed two activity bands in all *H. rhodopensis* samples (Figure 7). In contrast to SOD, the relative total CAT activity in leaves was highest in the control, followed by the LD1 sample (Figure 7 right). In the LD2 sample, the enzyme activity was significantly suppressed (about seven times weaker). Further dehydration resulted in a gradual increase in CAT activity to 59, 73, and 77%, respectively, of that of the control. Both isoenzymes were responsible for the recorded changes in total activity. In the first two dehydration steps, root total CAT activity decreased to 86 and 81% of that of the control (*p* ≥ 0.05), but isoenzyme 2 activity was stimulated by 61 and 22%, respectively. At the third step (RD3), the decrease was about three-fold, while in the air-dry sample, there was a return to the control level due to upregulation of isoenzyme 2 (about a 50% increase). In the roots of rehydrated plants, the activity of isoenzyme 1 and isoenzyme 2 was 94% and 268% higher than that in roots of the control plants, leading to a significant increase in the total CAT activity by 153%. In general, the total CAT activity in leaves was significantly higher than in roots (by about three- to seven-fold and by 21% in rehydrated plants), except for the D2 state, where leaf CAT was 28% less active than CAT in roots. 

The GR isoenzyme profile of *H. rhodopensis* was represented by four common isoforms. Two additional, fastest-moving enzyme isoforms (5 and 6) were visible only in the roots (Figure 7 left). The total leaf GR activity was significantly stimulated in LD2 and LD4 samples compared to the well-hydrated control (by 26 and 36%, respectively) due to increased activity of isoform 2 (by 5.5 and 6 times, respectively) and isoform 3 (by 3.3-fold in both samples) (Figure 7). Leaf enzyme activity was 47% lower than in the control at the initial step of dehydration, but the activity of isoforms 2 and 3 was higher by about 440 and 120%. In LD3 and LR, as well as in the control, isoforms 1 and 4 were significantly more active than the other 2 isoforms and similar total GR activity was determined in these samples. However, for LD3 and LR, isoform 1 activity was higher by 90 and 23%, respectively, than that of the control. In roots, there was an increase in total GR activity in RD2 and RD3 by 18 and 30% (*p* ≤ 0.05), respectively, compared to the control (Figure 7 right). Before and after these steps of treatment, total root GR activity did not differ significantly from the control. Despite this, in RD1, the activities of isoforms 3, 4, and 5 were increased by 84, 100, and 32%, respectively. Isoform 3 activity was 92 and 58% higher in RD4 and rehydrated roots, respectively, and isoform 4 was about 64% more active in the rehydrated sample than in the control (Figure 7). All root samples, except RD4, demonstrated significantly higher total GR activity (150, 295, 141, 183, and 175%, respectively) than their corresponding leaf samples, whose activity was taken as 100%.

Fourteen glutathione S-transferase (GST) isoenzymes were distinguished in the leaf samples of *H. rhodopensis* (Figure 7 left). The intensity of most GST activity bands was highest in the control, resulting in a significantly lower total GST activity in all but one sample. The total GST activity in LD4 was not different from that of the control, due to the increased activity of isoforms 3, 4 and 11 (by 32, 445, and 20%, respectively). Notably, leaf isoform 4 was upregulated throughout the course of dehydration (67, 172, 226, and 445% increase in activity, respectively). In addition to the 14 GST isoforms observed in the leaves, 3 additional, faster-moving isoenzymes (15–17) were clearly visible in root samples (Figure 7 left). The total GST activity in RD2 and in the roots of rehydrated plants was similar to that of the control, but some specific isoenzymes were activated in response to these treatments. The most responsive in RD2 were isoenzymes 6, 8 and 10 (115, 300, and 48% higher activity), while isoenzymes 6, 8, 10 and 17 had higher activity by about 28, 115, 23, and 140%, respectively, and were more active after rehydration. Although the total enzyme activity in RD4 was significantly lower than in RC, the activity of isoform 16 and especially isoform 17 in the RD4 sample was significantly increased (by 32 and 140%, respectively). In the control, D2, and the rehydrated sample, total GST activity in roots was significantly higher than in leaves, mainly due to the high activity of root isozymes 9–13. In the D1, D3, and D4 states, however, leaf GST activity was not significantly different from root GST activity (Figure 7 right).

### 2.5. The Alterations in Stress-Induced Proteins (Dehydrins, ELIPs, sHSPs) in Leaves and Roots during Desiccation

**Figure 8:**
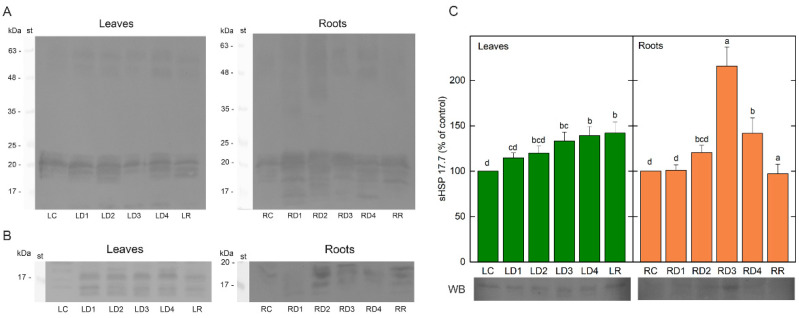
Western blots (WB) of dehydrins (**A**), ELIPs (**B**), and sHSP 17.7 abundance and WB (**C**) of leaves and roots of *H. rhodopensis*. Well-hydrated (LC; RC), dehydrated to different extents (LD1–LD4; RD1–RD4), and fully rehydrated (LR, RR) plants, according to Table 1. A total of 25 μg of protein was applied per lane.

The protein pattern of dehydrins, hydrophilic proteins belonging to the LEA2 group of stress-induced proteins, was monitored in the course of dehydration, as well as after rehydration of *H. rhodopensis* leaves and roots. Immunoblots showed the presence of several signals in the range of 15–63 (63, 48, 18–22, and 15) and 16–60 (60, 50, 42, 35, and 16–22) kDa in leaves and roots, respectively (Figure 8A). The protein profile was very similar in both types of samples. Dehydrins could be distinguished in control leaves and roots, especially in the low molecular weight area (15—22 kDa). Dehydration enhanced dehydrin abundance in both leaves and roots, and this enhancement was much more pronounced in root samples, where new dehydrin bands with molecular weights around 42 and 50 kDa appeared. Low molecular band dehydrins were also much more pronounced in roots compared to leaves. Rehydration of plants somewhat lowered the protein abundance of dehydrins, but not to an important extent. 

The protein content of ELIPs, part of the LHC a/b-binding proteins superfamily with photoprotective functions, was investigated in the leaves and roots of *H. rhodopensis* during dehydration as well after rehydration of plants by Western blot using antibodies raised against ELIPs. After SDS-PAGE separation and immunoblots, we detected various bands with apparent molecular weights of 14–19 and 16–19 kDa in leaves and roots, respectively (Figure 8B). The protein profile differed in both types of samples and ELIPs content/expression were more pronounced in leaf samples. Three main bands and at least two additional minor ones were monitored in leaves, while in roots only three bands were present. The proteins could be distinguished in both control leaves and roots. Dehydration led to a raised ELIP abundance in both types of samples compared to their respective controls. During rehydration, ELIPs were still present in both leaves and roots, but with lower signal intensity.

The sHSP 17.7 content in leaves and roots of *H. rhodopensis* during dehydration and after rehydration was monitored by Western blot. Dehydration of plants gradually increased the content of sHsp 17.7 in leaf samples, reaching the highest value of 140% in D4 (Figure 8C). Moderate dehydration (D1) did not affect sHSP 17.7 abundance in roots. The subsequent decrease in WC led to raised levels of the investigated protein, especially in D3 where the content of sHSP 17.7 increased two-fold compared to the control. During rehydration, the levels of sHSP 17.7 remained high in leaves, while in roots, the protein abundance decreased to reach control values.

## 3. Discussion

In the present study, we investigated for the first time the drought-induced changes in the defense mechanisms and alterations in the structure of the roots that correspond to the survival of complete desiccation. We also compared the defense mechanisms in roots to that in leaves, which were previously shown to be essential factors [7].

Oxidative stress is one of the most deleterious consequences of water deficit in plants [28]. As a homoiochlorophyllous resurrection plant, *H. rhodopensis* preserves chlorophyll during dehydration, which is a source of ROS production. Thus, an active antioxidant system is crucial for survival under harsh drought conditions. One of the significant differences between desiccation-sensitive and resurrection plants is the ability of the antioxidant enzymes in the latter to maintain the native state and to detoxify ROS in dry conditions even at RWC of <10% [29]. Regardless of the individual differences among resurrection plant species, they have in common highly effective enzymatic and non-enzymatic antioxidant systems [11]. In general, we observed that the antiradical and antioxidant activities remained high during desiccation in both leaves and roots, but some differences in their response were determined (Figure 4). The total antioxidant activity was higher in leaves compared to roots. Overall, the content of non-enzymatic antioxidants remained close to control levels or increased in leaves and roots at different degrees of desiccation and after rehydration. 

It is supposed that specific UV-B photoreception is involved in the stimulation of flavonoid biosynthesis as the action spectrum for flavonoid synthesis exhibits the maximum at 295 nm with little effectiveness shown for wavelengths >320 nm [30]. There is much evidence in the literature that many plants express photoreceptors, including several phytochromes, cryptochromes, and phototropins, which are found not only in the aerial parts of plants but also in the roots [31]. This indicates that the root system possesses the physiological ability to respond to broad light wavelengths, intensities, and directions [32]. From this point of view, it could be supposed that environmental stress would influence not only the UV-absorbing compounds in leaves, but also in the roots. Our data showed that during the desiccation of the resurrection plant *H. rhodopensis*, the alteration of the content of UV-absorbing compounds was observed for both leaves and roots. The content of UV-absorbing compounds (Figure S3) and total flavonoids (Figure 4) increased in leaves, while a significant enhancement in roots was observed at D3. The electron-dense dark deposits in the root cells resembling tannin [33] correspond to the accumulation of these UV-absorbing compounds. Nevertheless, further analyses are required to determine the exact chemical composition of the tannin-like deposits. We recorded an increase in anthocyanins content at D1 and after rehydration in leaves and roots, as well as in D3 in leaves (Figure 5). A high level of anthocyanins during the desiccation of HDT species is important for masking chlorophyll and thus reducing light absorption and ROS production [14]. The Asc amount remained high during desiccation and after rehydration of leaves and roots, but the highest values were also determined at D3 (Figure 5). Similarly, it has been shown that after the initial decline in ascorbate content in the roots of *Xerophyta viscosa* at 80% RWC, it increased almost to the control level and remained constant upon further drying [27]. The initial drop in Asc is suggested to be due to its utilization in quenching elevated levels of ROS caused by initial water loss. The authors propose that the elevated Asc content in roots and leaves is maintained during drying and early rehydration by a combination of both de novo synthesis and regeneration of Asc by APX, which itself retains the ability to remain active [3,5,27]. In addition to its involvement in the ascorbate-glutathione cycle scavenging of H_2_O_2_, Asc can directly quench activated oxygen species and acts as a signaling molecule involved in the regulation of the plant’s response to stress conditions [34,35]. Thus, maintaining a high content of non-enzymatic antioxidants in *H. rhodopensis* leaves and roots during desiccation may play an important role in coping with possible oxidative damage and may provide protection at the onset of rehydration.

Many studies confirm the importance of antioxidant enzymes in ROS detoxification [6,7]. However, considerable variation in the activity of antioxidant enzymes is observed, depending on the plant genotype, environment, severity, and duration of stress [36,37]. It is concluded that each resurrection plant has its own specificity in antioxidative response [5]. For example, it has been found that SOD activity decreased, but CAT and peroxidase (APX) activities showed no significant differences during the desiccation of *Tripogon spicatus* [38]. However, SOD, APX, and GST remained overexpressed throughout the desiccation of *Selaginella bryopteris* [39]. Significant enhancement in APX and GR activities at the beginning of the desiccation of *Ramonda nathaliae* is reported but it was reduced during further water loss to nearly the level of the control in fully desiccated leaves [40]. The activity of APX, GR, and GST increased upon desiccation of *H. rhodopensis*, reaching a maximum in the air-dry state [7]. SOD constitutes the first line of defense against ROS, catalyzing the disproportionation of superoxide radicals to molecular oxygen and H_2_O_2_ [41]. The accumulation of H_2_O_2_ is prevented by the activities of CAT, peroxidases, and GR. GST has multiple functions, including the glutathione-dependent reduction of organic hydroperoxides formed during oxidative stress [42]. Our present results showed that with the exception of CAT, the activities of SOD, GR, and GST were higher in well-hydrated roots compared to leaves. Overall, the relative total antioxidant enzyme activity remained high during the desiccation of both leaves and roots (Figure 7). It should be mentioned that SOD and GR activities were higher in roots than in leaves upon desiccation, but CAT was higher in leaves (Figure 7). In addition, in most of the treatments, the activity of specific isoenzymes was upregulated. For example, in roots, SOD isoforms 1 and 5 were significantly activated at RD1 and RD4. The activity of SOD isozymes 3 and 4 in RD3 was increased by about 150% compared to the control. In leaves, GR isoforms 2 and 3 were upregulated in LD1. Moreover, some isoforms such as GST isoform 4, were upregulated throughout the course of dehydration. An increase in the activity of a particular isoform of any antioxidant enzyme could be even more effective in protecting against oxidants than the enhanced total enzyme activity, due to the action of that isoform in a specific cellular compartment [43]. Furthermore, two GR isoforms and three GST isoforms were visible only in the roots (Figure 7). While the relative total activity of the studied antioxidant enzymes was close to the control after the rehydration of leaves, it remained increased in roots. Thus, the upregulation of the activity of individual enzyme isoforms and maintenance of a high total antioxidant enzyme activity in desiccated leaves and roots of *H. rhodopensis*, as well as after 6 days of rehydration, indicated their essential role in protecting plants from oxidative damage under these conditions. It has been suggested that antioxidants accumulated upon desiccation are reserves that can be used in the early stages of rehydration during the recovery of metabolism [6,44]. A decrease in the activity of SOD, CAT, APX, and GR in the initial stages of the root desiccation of *Xerophyta viscosa* is observed, followed by relatively constant activity during further drying to the air-dry state [27]. Our data, however, showed higher activity of antioxidant enzymes during the desiccation of *H. rhodopensis* roots compared to *Xerophyta viscosa*. This response may be species-specific as *H. rhodopensis* is a homoiochlorophyllous plant, whereas *Xerophyta viscosa* is a pokilochlorophysllous resurrection plant. Furthermore, the data confirm that antioxidant enzymes were not denatured by desiccation of the root tissue, and maintenance of antioxidant potential in roots during desiccation in these resurrection plants is important for protection against ROS. NO is a signaling molecule, but also a free radical (reactive nitrogen species, RNS) that can initiate reversible protein Cys-nitrosylation and irreversible Tyr-nitration, thus affecting protein and enzyme function [45,46]. In addition, it can form adducts with glutathione, thus actively interacing with the redox status of the cells [47,48]. The NO adduct of glutathione is S-nitrosoglutathione (GSNO) which can either donate or liberate NO, thus representing a stored form of NO [49]. GSNO is eliminated by GSNO reductase, which requires S-(hydroxymethyl)glutathione (a spontaneous glutathione formaldehyde adduct) as a source of reducing power to reduce nitrogen nuclei to ammonia [50]. Thus, the control over NO metabolism highly depends on the redox status and the capacity of the antioxidative system. Since specific antioxidative enzymes such as GR and non-enzymatic antioxidative constituents such as Asc seem to be inducible in the roots upon severe desiccation, the effective elimination of NO in the roots is suggested to be a result of the hardening of the antioxidative capacity and the restoration of the redox status. Increased NO production in the roots is a widely studied response of plants under drought stress [51]. The increase in NO production upon drought stress contributes to stress tolerance, as the application of an exogenous NO source also improves the drought tolerance of plants [52,53,54]. Although NO is also involved in ABA-induced stomatal closure [55,56], the role of drought-induced NO production in roots has not been fully revealed yet. Thus, the massive increase in the NO signal of *H. rhodopensis* roots upon the appearance of drought stress should be considered a typical drought stress response. Nevertheless, the decline in NO accumulation under severe water loss in *H. rhodopensis* roots should be kept specific to the desiccation-tolerant taxon that could also be responsible for retaining the viability of the hardly studied roots of resurrection plants.

Accumulation of stress-induced proteins is one of the main defense mechanisms in resurrection plants upon drought stress [11,57]. However, similar to antioxidants, most of the studies were performed on leaves. Proteomic analysis carried out in *Selaginella bryopteris* roots showed that out of 59 identified spots, 58 protein spots are found to be upregulated during desiccation, including many proteins with antioxidative properties [39]. It has been shown that *Selaginella bryopteris* roots were able to cope with dehydration by maintaining their protein synthesis machinery in a stable state during dehydration/rehydration. In fact, similar proteome changes in roots and fronds during dehydration and rehydration are observed. A significant number of proteins that appear during desiccation in leaves correspond to the alterations in carbohydrate metabolism [58]. These alterations contribute to the removal of the starch granules from chloroplasts and the accumulation of carbohydrates in the secondary vacuoles [59]. However, the removal of starch granules is not a universal process in all cells of *H. rhodopensis*, since in the roots, starch granules remained in the cells of the central cylinder. Abiotic stress such as drought (in fact air-drying) caused enhanced suberization and lignification of the exodermis and endodermis cells of maize roots [60]. Under desiccation conditions, the same layers of the roots of *H. rhodopensis* were affected in our study (Figure 2).

Recently, shotgun proteomics showed the enhanced protein abundance of dehydrins, ELIPs, and HSPs in leaves of *H. rhodopensis* during desiccation [61]. The increased protein content of dehydrins was monitored during drought- and freezing-induced desiccation of Balkan’s resurrection species *H. rhodopensis*, *Ramonda serbica*, and *Ramonda nathaliae* [58,62]. Dehydrins have chaperone-like functions in plant cells related to the protection of proteins and membrane stabilization during stress, but they also act as ROS scavengers [63,64]. Our present results showed that desiccation of *H. rhodopensis* induced enhanced dehydrin abundance in both leaves and roots, and this enhancement was much more pronounced in root samples, where new dehydrin bands appeared. Similarly, significantly higher dehydrin gene expression in roots in comparison with leaf tissues in *Craterostigma plantagineum* is observed [16]. It has been found that desiccation induces transient phosphorylation of the abundantly expressed protein CDeT6-19, belonging to the group of dehydrins in *Craterostigma plantagineum* leaves and roots [65]. The phosphorylation of CDeT6-19 was suggested as an important prerequisite for the function of this protein. It is concluded that phosphorylation and dephosphorylation of proteins are part of the desiccation tolerance mechanism of *Craterostigma plantagineum* and *H. rhodopensis* [61,65].

Furthermore, homoiochlorophyllous desiccation-tolerant species upregulate ELIPs that are thought to bind chlorophyll, preventing photooxidative damage [8]. Immunoblot analysis showed that ELIPs could be distinguished in both well-hydrated *H. rhodopensis* leaves and roots. Dehydration resulted in increased ELIP abundance in both sample types compared to controls, but their expression was more pronounced in leaf samples. The expression of ELIPs during the leaf and root desiccation of *C. plantagineum* revealed low or undetectable ELIP expression in the well-watered state, but they were among the most highly expressed genes during drying and recovery [16]. ELIPs had significantly lower expression in root tissues under desiccation and they were still highly expressed 48 h post-rehydration, suggesting their protective role during the recovery of photosynthetic apparatus. ELIPs were also detected in leaves in *H. rhodopensis* during freezing-induced desiccation, as well after the rehydration of dried plants [66,67].

In addition to dehydrins and ELIPs, we investigated the changes in sHSP 17.7 content in the leaves and roots of *H. rhodopensis* during dehydration and after rehydration. Desiccation resulted in a gradually increasing content of sHsp 17.7 in leaves and it remained high after rehydration. The highest abundance of sHSP 17.7 in roots was observed in D3 and it was much higher than in leaves but declined to the control level after rehydration. Plant sHSPs are constitutively expressed in plant cells at low concentrations and strongly induced in response to different stress factors [68]. They act as ATP-independent molecular chaperones, preventing the irreversible aggregation of denatured proteins [69]. Fourteen sHSP isoforms are detected in *H. rhodopensis* leaves under severe dehydration in response to high-temperature stress [70]. Transcripts encoding HSPs are abundantly expressed under unstressed conditions in vegetative tissues of *H. rhodopensis*, suggesting the readiness of the plant to combat stress [71]. The importance of sHSPs for the maintenance of protein stability during the desiccation of the resurrection plant *Boea hygrometrica* was confirmed [72]. Out of 25 sHSP genes identified, only six genes are upregulated during desiccation and rehydration, and the majority of them were constitutively expressed. In addition, the constitutive expression of plant sHSPs in the roots and leaves of *Craterostigma plantagineum* is observed [73]. The polypeptides, related to HSP17.6 and HSP17.9, accumulated in desiccated roots. Moreover, HSP70 is found to be upregulated in *Selaginella. bryopteris* roots during desiccation and rehydration [39]. Thus, the expression of sHSPs could contribute to the ability of resurrection plants to withstand cytosolic dehydration, protecting sub-cellular components in specific tissues.

## 4. Materials and Methods

### 4.1. Plant Material and Experimental Design

*H. rhodopensis* Friv. tufts were initially collected from the Rhodope Mountains, Bulgaria, and vegetative propagation was performed in pots with peat soil (Stender, Schermbeck, Germany) under ex situ (outdoor) environmental conditions. After propagation, rosettes were cultivated at least for one year prior to the experiments. Plants were transferred to a FytoScope FS 130 climatic chamber (Photon Systems Instruments, Drásov, Czech Republic) and kept at 25/18 °C (day/night temperature), 60% relative humidity, and irradiance of 25 μmol photons m^−2^ s^−1^ for two weeks with a photoperiod of 12 h. Then plants were subjected to drought stress by withholding irrigation and desiccated to an air-dry state. Samples were taken from mature plants as follows: from well-hydrated control leaves (LC) and roots (RC) and after their dehydration to different extents to an air-dry state (LD1-LD4–leaves, RD1-RD4–roots), as well after 6 days of rehydration of dry plants (LR–leaves, RR–roots). Leaves and roots of *H. rhodopensis* reached moderate desiccation (LD1 and RD1, respectively) in 15 days, and the air-dried state (LD4 and RD4, respectively) after 22 days. Samples between these two stages of desiccation, LD2-LD3 and RD2-RD3, were collected on the 17th and 18th days, respectively. At each time point, leaves and roots from the same tuft (with 4–6 individual plants) were used for analysis. Plants were rehydrated in a modified desiccator, where the desiccant at the bottom was replaced by water, providing permanent high humidity by a water pump. It is important to note that tufts have adventitious roots developing from the horizontally growing rhizome. However, for simplicity, in the Results and in the Discussion, we only refer to them as roots of *H. rhodopensis*.

### 4.2. Determination of Water Content (WC)

The WC of leaves and roots was determined gravimetrically by weighing them before and after oven-drying at 80 °C to a constant mass and expressed using the following equation: WC = (FM − DM)/DM, where FM—fresh mass, and DM—dry mass. The WC was calculated as g H_2_O g^−1^ DM.

### 4.3. Nitric Oxide (NO) Determination

*H. rhodopensis* root tips were collected at different stages of desiccation. After all soil contaminations were removed by a quick but careful cleaning using a bristle to avoid any alteration in the hydration status, intact root tip segments were collected and stained for 30 min in 5 mM DAF-FM diacetate; (Sigma, Saint Louis, MO, USA). Fluorescence of the DAF-FM NO condensate was detected using a Nikon Eclipse 80i epifluorescent microscope mounted with an FITC filter system (excitation maximum: 475 nm; emission filter: 530–543 nm). As for the negative control/basis of normalization, the unstained root tips of identical hydration status were applied. Fluorescence micrographs were taken at 40× magnification by a Spot 7.4 Slider camera (SPOT Imaging, Sterling Heights, MI, USA) and recorded at identical exposure times. For image processing and arithmetic calculations of pixel intensities, ImageJ software (V 1.8.0) was applied. Average pixel intensity of the unstained root segments was subtracted from that of the DAF-FM NO signal after baseline correction of the background noise. Data represent the mean (±SE) of *n* = 9.

### 4.4. Microscopy

*H. rhodopensis* root segments were collected at different stages of desiccation and cleaned as described at NO detection. To analyze the structure, samples were taken at the branching point of the primary adventitious root. Root segments 2–3 mm in length, 5 mm above the root tip, were collected in fresh-made 70 mM K-Na phosphate (pH 7.2), 2.5% (*V*/*V*) glutaraldehyde solution for fixation for at least 3 h. Rinsing of the primary fixative with buffer, post-fixation in osmium-tetroxide, rinsing of the post-fixative in buffer, dehydration in ethanol, embedding in Durcupan ACM (Fluka, Buchs, Switzerland), and semithin (1 µm) sectioning were carried out as described previously [74]. Sections were stained with toluidine blue and studied using an Olympus BH2-RFCA light microscope (Olympus, Tokyo, Japan). Digital light microscopic images were captured using a Nikon COOLPIX 950 (Nikon, Tokyo, Japan) camera. After semithin sectioning, 70 nm ultrathin sections were cut from the same root sections and contrasted with lead citrate and uranyl-acetate before being analyzed with a JEM-1011 (JEOL, Tokyo, Japan) transmission electron microscope equipped with a Morada digital camera (Olympus, Tokyo, Japan) as in [75].

### 4.5. Free Radical Scavenging Activity Assay

The free radical scavenging activity was measured according to Brand-Williams et al. [76]. 1,1-diphenyl-2-picrylhydrazyl (DPPH) was used as the source of free radicals. A total of 100 mg of leaf and root material was ground with 2 mL 80% ethanol and the homogenate was centrifuged at 13,000× *g* at 4 °C for 30 min. Briefly, a freshly prepared DPPH reagent (1.99 mL) and extract solution (0.01 mL) were mixed, and the absorbance was measured at 515 nm (UV-VIS Specord 210 Plus, Analytic Jena, Jena, Germany). For the blank, methanol was used. The free radical scavenging activity was calculated from a standard curve made with known concentrations of Trolox. The results were presented on a dry weight basis as a mean (± SE) of *n* = 12.

### 4.6. Ferric-Reducing Antioxidant Power Assay

Total antioxidant activity was investigated using the ferric-reducing antioxidant power (FRAP) assay [77]. This procedure involved the reduction of ferric ions (Fe^3+^) to ferrous ions (Fe^2+^) in a blue-colored complex in the presence of bioactive compounds (antioxidants). A total of 100 mg of leaf and root material was ground with 2 mL 80% ethanol and the homogenate was centrifuged at 13,000× *g* at 4 °C for 30 min. Briefly, freshly prepared FRAP reagent and extract solution (0.05 mL) were mixed, and the absorbance was measured at 593 nm (UV-VIS Specord 210 Plus, Analytic Jena, Jena, Germany), using the FRAP solution as a blank. The antioxidant potential of samples was calculated from a standard curve plotted using known concentrations of FeSO_4_·7H_2_O. The results were presented on a dry weight basis as a mean (±SE) of *n* = 12.

### 4.7. Determination of Total Flavonoid Content

Total flavonoid content was determined as previously described [78]. The method is based on the formation of chelate complexes of flavonoids with AlCl_3_ and interaction of the chelates with NaNO_2_ and NaOH, in which a red-colored complex is formed. A total of 100 mg of leaf and root material was ground with 2 mL 80% ethanol and the homogenate was centrifuged at 13,000× *g* at 4 °C for 30 min. The 2 mL reaction mixture contained 200 μL extract, 1.28 mL ddH_2_O, 60 μL 5% (*w*/*V*) NaNO_2_, 60 μL 10% (*w*/*V*) AlCl_3_, and 400 μL 1M NaOH. The absorbance was measured at 510 nm (UV-VIS Specord 210 Plus, Analytic Jena, Jena, Germany), using 80% ethanol as a blank. Flavonoid content was determined from a standard curve plotted using known concentrations of rutin. The results were presented on a dry weight basis as a mean (± SE) of *n* = 12.

### 4.8. Determination of Ascorbate Content

An amount of 100 mg of leaf and root tissue was ground with 2 mL cold 6% trichloroacetic acid (TCA) and centrifuged at 15,000× *g* for 20 min at 4 °C [79]. To 0.2 mL of the supernatant, 0.6 mL 0.2 M K-phosphate buffer (pH 7.4), 0.2 mL dd H_2_O, 1 mL 10% (*V*/*V*) TCA, 0.8 mL 42% (*V*/*V*) H_3_PO_4_, 0.8 mL 4% (*w*/*V*) 2,20-dipyridyl, and 0.4 mL 3% (*w*/*V*) FeCl_3_ were added. All samples were incubated for 40 min at 42 °C, and their absorbance was read at 525 nm using a UV-VIS Specord 210 Plus spectrophotometer (Analytic Jena, Jena, Germany). The results were presented on a dry weight basis as a mean (± SE) of *n* = 6.

### 4.9. Anthocyanins and UV-Absorbing Compounds

Leaf and root material (100 mg) was homogenized with 6 mL of medium containing ethanol/HCl/dH_2_O (79/1/20, *V*/*V*/*V*). The resulting homogenate was centrifuged at 10,000× *g* for 15 min at 4 °C. The absorbance of the clear extract was registered at 535 and 653 nm using a UV–VIS spectrophotometer (UV-VIS Specord 210 Plus, Analytic Jena, Jena, Germany) to determine the content of anthocyanins, calculated by the formula *A*_535_ − 0.24 × *A*_653_ [80]. The absolute values of anthocyanins were calculated using a molar extinction coefficient 33,000 M^−1^ cm^−1^ [81] and presented on a dry weight basis as a mean (±SE) of *n* = 6 (μmol anthocyanins g^−1^ DW). 

For registration of UV-absorbing compounds after centrifugation, the clear extract was diluted 25-fold by the homogenizing medium and absorbance spectra were recorded in the spectral region of 240–500 nm. Additionally, the absorbance was registered at 280 and 336 nm. 

### 4.10. Protein Extraction, nPAGE, and Antioxidant Enzyme Activity Staining

After grinding in liquid nitrogen, plant material (0.5 g FW) was homogenized in ice-cold 50 mM potassium-phosphate buffer (PPB, pH 7.8) containing 10 mM KCl, 1 mM EDTA, 1.25 mM PEG 4000, 0.5 M sucrose, 20 mM ascorbic acid, 10 mM dithiothreitol (DTT), 0.1% (*V*/*V*) Triton-X 100, 2 mM PMSF, and 2% (*w*/*V*) Polyclar AT [43]. After centrifugation for 30 min at 15,000× *g*, 4 °C, total protein extracts were desalted on Sephadex G 25 mini columns. Eluted proteins were supplemented with sucrose (20% final concentration, *w*/*V*) and stored in aliquots at –80 °C. The protein concentration was calculated following the dye-binding assay [82]. Equal amounts of protein (15 µg) from the leaves of plants exposed to different treatments were subjected to discontinuous PAGE under non-denaturing, non-reducing conditions [83], but omitting SDS. Electrophoretic separation of proteins lasted 4–5 h at a constant current of 35 mA per gel. When nPAGE was finished, separate gels were stained for the activities of superoxide dismutase (SOD, EC 1.15.1.1), catalase (CAT, EC 1.11.1.6), glutathione reductase (GR, EC 1.6.4.2), and glutathione S-transferase (GST, EC 2.5.1.18). To visualize the bands with SOD, gels (10% polyacrylamide) were soaked in 0.1 mM nitroblue tetrazolium (NBT), 0.05 mM riboflavin, and 0.3% (*V*/*V*) tetramethyl ethylene diamine in 50 mM PPB (pH 7.8) for 20 min in the dark. Thereafter, the gels were submerged in dH_2_O and exposed to a light box for about 10 min [84]. For CAT activity staining, the gels (6.5%) were pretreated in 0.01% H_2_O_2_ for 10 min and incubated in a staining solution containing 1% (*w*/*V*) ferric chloride and 1% (*w*/*V*) potassium ferricyanide mixed in equal volumes during use [85]. The staining solution for GR isoenzyme patterns and activity determination consisted of 0.24 mM 3-(4,5-dimethylthiazol-2-yl)-2,5-diphenyltetrazolium bromide (MTT), 0.34 mM 2,6-dichlorophenolindophenol, 3.6 mM GSSG, and 0.4 mM NADPH in 250 mM Tris-HCI buffer (pH 7.8). The 7.5% gels were immersed in this solution for 1 h in darkness [86]. GST isoforms and activity were detected as described previously [87]. Briefly, the 10% resolved polyacrylamide gels, equilibrated in 100 mM PPB (pH 6.5) for 10 min, were transferred to a reaction mixture composed of 4.5 mM GSH, 1 mM 1-chloro-2,4-dinitrobenzene (CDNB), and 1 mM NBT in the same PPB buffer for 10 min at 37 °C. Further, the gels were incubated at room temperature in 100 mM Tris-HCl (pH 9.6) containing3 mM phenazine methosulphate (PMS) until they become uniformly blue, while the bands with GST activity were achromatic. After staining, the enzyme patterns were documented using the UVItec gel documentation system (Cambridge, UK) and analyzed using Gel-Pro32 Analyzer software (Media Cybernetics, Rockville, MD, USA). The intensity (activity) of each band (isoenzyme) resolved was measured as total integrated optical density (IOD), in arbitrary units. Each enzyme had more than one isoenzyme, and the sum of their IOD values was considered total enzyme activity for a particular treatment. The experiments to determine the pattern and activity of each enzyme were repeated at least three times. 

### 4.11. Total Leaf Protein Isolation, SDS-PAGE, and Western Blot

Total leaf proteins were extracted in the sample buffer [62] and their content was determined [82]. Isolated samples were separated on 12% (dehydrins) or 16% (ELIPs, sHSP 17.7) SDS-PAGE (SE260 Mighty Small II, Hoefer, Holliston, MA, USA) polyacrylamide gels [83], modified by adding 8.0% (*V*/*V*) glycerol to stacking and separating gels using a constant current of 20 mA per gel. Each lane contains 25 μg total leaf protein. The proteins were blotted on a nitrocellulose membrane for 90 min at a current of 1 mA cm^−2^ using semi-dry transfer (TE70X, Hoefer, Holliston, MA, USA). ROTI^®^Mark TRICOLOR (Carl Roth GmbH + Co. KG, Karlsruhe, Germany) pre-stained protein standard was used for monitoring transfer efficiency. Blots were probed with primary antibodies against ELIPs (AS06 147A, Agrisera, Vännäs, Sweden), dehydrin K-segment (AS07 206A, Agrisera, Vännäs, Sweden), and Hsp 17.7 (AS07 255 Agrisera, Vännäs, Sweden). Horseradish peroxidase-conjugated goat anti-rabbit secondary antibody was used (AS09 602, Agrisera, Vännäs, Sweden). The resulting bands were visualized by color reaction for sHsp 17.7 (6 mg DAB in 10 mL 50 mM (*w*/*V*) Tris-HCl, pH 7.6, 10 μL 30% (*V*/*V*) H_2_O_2_) or chemiluminescence on X-ray Blue films for dehydrins and ELIPs (Carestream Dental LLC, Atlanta, GA, USA). Membranes and films were scanned using an Epson Perfection V850 PRO scanner (Seiko Epson Corporation, Suwa, Japan) and densitometry was made by Gel-Pro Analyzer software (Media Cybernetic, Rockville, MD, USA). The experiments to determine the pattern or content of each protein were repeated four times.

### 4.12. Statistical Analysis

Leaves and roots for biochemical analysis were sampled from one tuft at each sampling point. The values obtained for each of the investigated parameters for leaves and roots in a well-hydrated state, during desiccation to an air-dry state, and after 6 days of rehydration were compared by the Fisher least significant difference (LSD) test or Tukey-Kramer test at *p* ≤ 0.05 following one-way ANOVA. A statistical software package (StatGraphics Plus, version 5.1 for Windows, The Plains, VA, USA) was used. 

## 5. Conclusions

Our study showed that maintaining a high content of non-enzymatic antioxidants and antioxidant enzyme activity in leaves and roots during the final stages of desiccation play an important role in coping with oxidative damage. They may provide protection at the onset of rehydration, thus contributing to the successful recovery of dry plants. In addition, the activity of specific isoenzymes was upregulated at different extents of desiccation. Moreover, desiccation of *H. rhodopensis* induced enhanced abundance of dehydrins, ELIPs, and sHSP 17.7 in both leaves and roots, indicating their importance in the acquisition of desiccation tolerance. However, we also found some differences in the response of leaves and roots to desiccation. Starch granules that eliminate in the mesophyll cells upon desiccation remain persistent in the cells of central cylinders in the roots. Regarding the antioxidative defense, SOD and GR activities were higher in the roots than in leaves upon desiccation, whereas CAT was higher in leaves. Furthermore, two additional GR and three additional GST isoforms were visible only in the roots. The enhancement in dehydrin abundance was much more pronounced in roots, while ELIPs increased more in leaves. The massive increase in the NO signal of *H. rhodopensis* roots upon the appearance of drought stress should be considered a typical drought stress response. Nevertheless, the decline in NO accumulation under severe water loss in *H. rhodopensis* roots should be kept specific to the desiccation-tolerant taxon that could also be responsible for retaining the viability of the hardly studied roots of resurrection plants. Data presented contribute to a better understanding of protective mechanisms in roots during the desiccation of the resurrection plant *H. rhodopensis*, but more research on the root tissues of different resurrection plants is needed to elucidate the desiccation tolerance strategies of whole plants.

## Figures and Tables

**Table 1:** Water content (WC) of control leaves (LC) and roots (RC) during desiccation to different extent (LD1–LD4; RD1–RD4) and after 6 days of rehydration (LR; RR).

Variant Leaves	WC of Leaves (g H_2_O g DW^−1^)	Variant Roots	WC of Roots (g H_2_O g DW^−1^)
LC	4.96 ± 0.08 a	RC	3.82 ± 0.07 b
LD1	2.16 ± 0.20 d	RD1	1.14 ± 0.09 ef
LD2	1.30 ± 0.14 ef	RD2	1.30 ± 0.06 ef
LD3	0.88 ± 0.16 f	RD3	1.43 ± 0.01 e
LD4	0.22 ± 0.03 g	RD4	0.35 ± 0.01 g
LR	4.10 ± 0.14 b	RR	3.23 ± 0.30 c

## Data Availability

All datasets are contained within the article and in the Supplementary Materials.

## Supplementary Materials

The following supporting information can be downloaded at: https://www.mdpi.com/article/10.3390/plants12152834/s1, Figure S1: Appearance of *H. rhodopensis* leaves and roots in well-hydrated and air-dry state; Figure S2: Nitric oxide accumulation in the apical zone of roots detected by the autofluorescence of DAF-FM NO conjugates; Figure S3: Changes in the absorbance at 280 nm and 336 nm of the extracts from leaves and roots from *H. rhodopensis*.
