# Supplementary material for: Antioxidative Defense, Suppressed Nitric Oxide Accumulation, and Synthesis of Protective Proteins in Roots and Leaves Contribute to the Desiccation Tolerance of the Resurrection Plant Haberlea rhodopensis
Source: Plants (Basel). 2023 Jul 31;12(15):2834. doi: 10.3390/plants12152834 (PMC10421438; doi:10.3390/plants12152834)
Supplement: Supplementary file 1 [file plants-12-02834-s001.zip › plants-2433215-supplementary.pdf]

Supplementary material

Antioxidative Defense, Suppressed Nitric Oxide Accumulation, and Synthesis of Protective Proteins in Roots and Leaves Contribute to the Desiccation Tolerance of the Resurrection Plant *Haberlea rhodopensis*

Katya Georgieva [1,*], Gergana Mihailova [1], Liliana Gigova [1], Antoaneta V. Popova [2], Maya Velitchkova [2], Lyudmila Simova-Stoilova [1], Máté Sági-Kazár [3,4], Helga Zelenyánszki [3,4], Katalin Solymosi [5] and Ádám Solti [3]

[1] Institute of Plant Physiology and Genetics, Bulgarian Academy of Sciences, Academic Georgi Bonchev Str., Building 21, 1113 Sofia, Bulgaria; gmihailova@bio21.bas.bg (G.M.); gigova01@gmail.com (L.G.); lsimova@mail.bg (L.S.-S.)

[2] Institute of Biophysics and Biomedical Engineering, Bulgarian Academy of Sciences, Academic Georgi Bonchev Str., Building 21, 1113 Sofia, Bulgaria; popova@bio21.bas.bg (A.V.P.); ma-yav@bio21.bas.bg (M.V.)

[3] Department of Plant Physiology and Molecular Plant Biology, Institute of Biology, ELTE Eötvös Loránd University, Pázmány Péter Sétány 1/C, H-1117 Budapest, Hungary; sagi.kazar.mate@ttk.elte.hu (M.S.-K.); helga.zelenyanszki@ttk.elte.hu (H.Z.); adam.solti@ttk.elte.hu (Á.S.)

[4] Doctoral School of Biology, Institute of Biology, ELTE Eötvös Loránd University, Pázmány Péter Sétány 1/C, H-1117 Budapest, Hungary

[5] Department of Plant Anatomy, Institute of Biology, ELTE Eötvös Loránd University, Pázmány Péter Sétány 1/C, H-1117 Budapest, Hungary; katalin.solymosi@ttk.elte.hu

* Correspondence: katya@bio21.bas.bg or georgieva.katya.m@gmail.com; Tel.: +359-2-979-2620

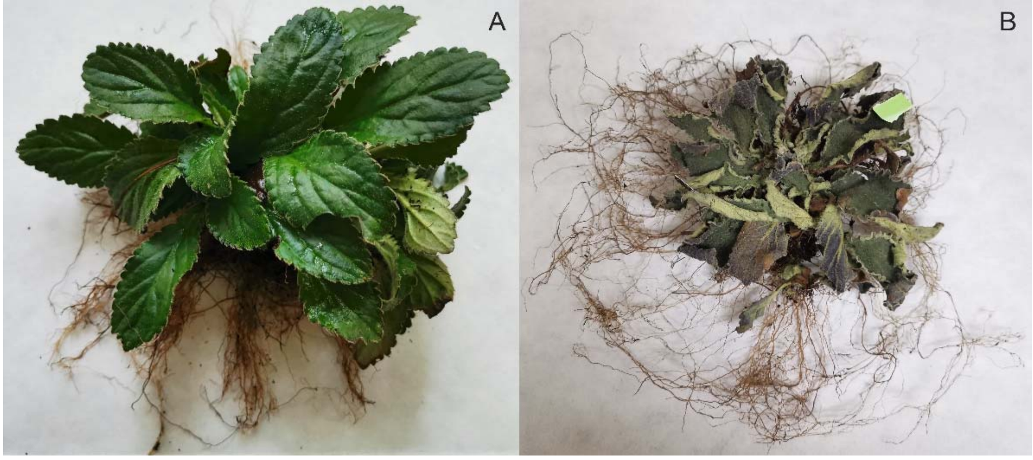

Figure S1. *H. rhodopensis* leaves and roots in well-hydrated (**A**) and air-dry state (**B**).

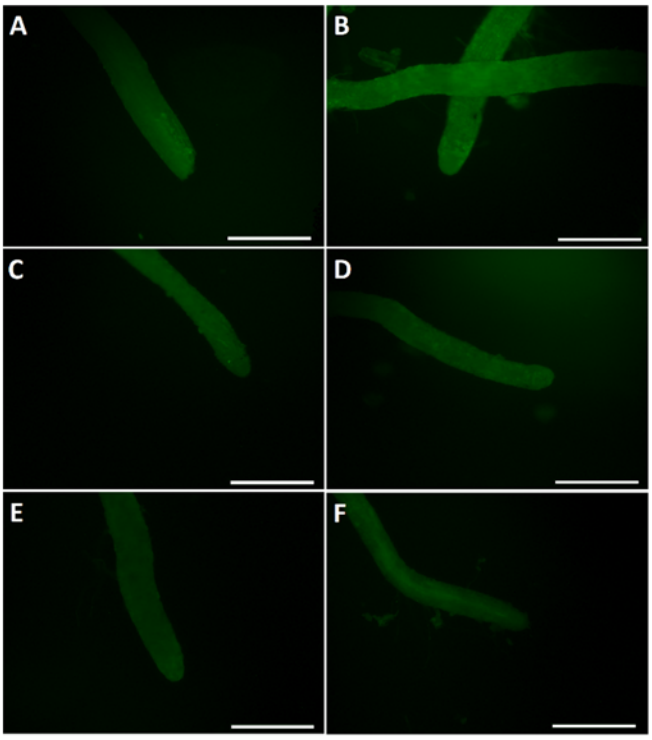

Figure S2. Nitric oxide accumulation in the apical zone of *H. rhodopensis* roots detected by the autofluorescence of 4-amino-5-methylamino-2′,7′-difluorofluorescein (DAF-FM) NO conjugates. A: RC; B: RD0; C: RD1; D: RD3; E: RD4; F: RR; where roots (R) were analyzed in well-hydrated control (C) stage, and during desiccation (D1-D4) and after 6 days of rehydration (RR), according to Table 1. RD0 is a supplementary sample that was taken at WC of 1.92±0.05 (g H_2O) (g DW^{-1}) as for an intermediate sample at the RC-RD1 transition. Bars are equal to 1 mm.

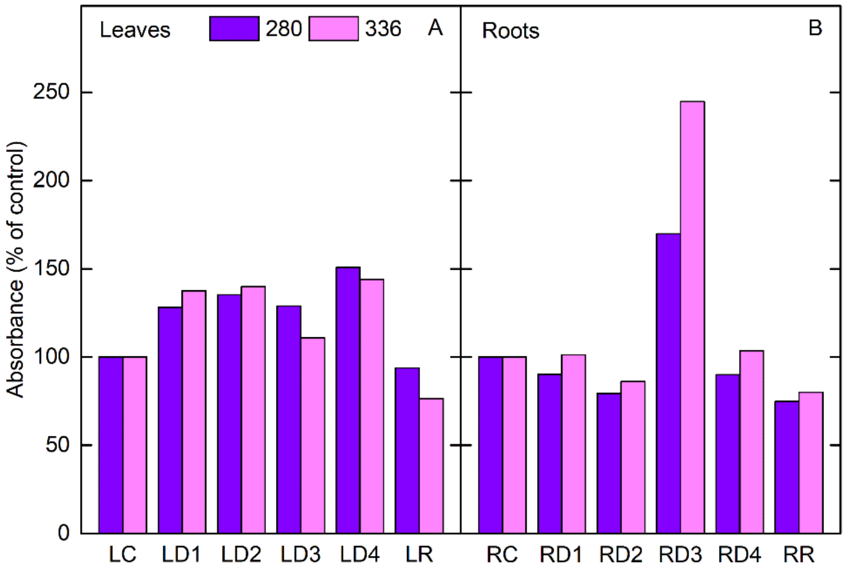

Figure S3. Changes in the absorbance at 280 nm and 336 nm of the extracts from leaves (L; **A**) and roots (R; **B**) from well-hydrated (LC, RC), dehydrated to different extent (LD1–LD4; RD1–RD4) and fully rehydrated (LR, RR) *H. rhodopensis* samples, according to Table 1. The values are presented as percentage (%) from the absorbance registered for fully hydrated plants.
